# Ultrastructural investigation of the posterior hyaloid membrane in posterior vitreous detachment

**DOI:** 10.1038/s41433-024-03407-4

**Published:** 2024-10-28

**Authors:** Thomas R. W. Nixon, Matthew J. Hayes, David Snead, Martin P. Snead

**Affiliations:** 1 Vitreoretinal Research Group, John van Geest Centre for Brain Repair, University of Cambridge, Cambridge, UK; 2 School of Clinical Medicine, University of Cambridge, Addenbrooke’s Hospital, Cambridge, UK; 3 University College London Institute of Ophthalmology, London, UK; 4 Department of Pathology, University Hospitals Coventry and Warwickshire NHS Trust, Coventry, UK

**Keywords:** Retinal diseases, Microscopy

## Abstract

**Background:**

Separation of the posterior hyaloid membrane (PHM) from the retina in posterior vitreous detachment (PVD) is a fundamental, but poorly understood, process underlying vitreoretinal disorders including retinal detachment and macular hole. We performed electron microscopy studies of the PHM after PVD to investigate its ultrastructure, associated cellular structures and relationship to the internal limiting membrane (ILM).

**Methods:**

Post-mortem human eyes were collected from recently deceased patients over 70 years of age. A posterior scleral button was trephined to identify PVD status, and the PHM and vitreous prepared for analysis with transmission and scanning electron microscopy.

**Results:**

Twelve eyes from six patients were collected. Seven eyes had PVD; five eyes had attached vitreous. PHM was isolated from seven of seven eyes with PVD. The PHM in eyes with PVD is a laminar lacy sheet, distinct from the disorganised fibres of vitreous gel. Eyes without PVD had vitreous encased in internal limiting membrane which had separated en bloc from the retina. Cells embedded in the PHM (laminocytes) were identified in five of seven eyes with PVD, with strands stretching into the membrane.

**Conclusions:**

PHM isolated from eyes with PVD is distinct from artefactual separation of the ILM from the retina during dissection. PHM is ultrastructurally distinct from vitreous gel and is a separate entity. The en face appearance of PHM is similar to that of ILM, suggesting that in PVD, PHM forms from separation of an inner layer of ILM. Laminocytes may play a role in the pathogenesis of vitreoretinal disease.

## Introduction

Posterior vitreous detachment (PVD) is the separation of the posterior hyaloid membrane (PHM) and vitreous from the retina and is the fundamental underlying pathophysiological process in many vitreoretinal disorders. Incomplete separation can lead to symptomatic vitreomacular traction. Abnormal separation can cause macular hole, or retinal tears with subsequent retinal detachment. Proliferation at the vitreoretinal interface can cause epiretinal membranes. Despite its importance to vitreoretinal disease, the process of PVD remains poorly understood.

Better understanding of the biological processes leading to PVD may allow prevention or early intervention in some of these sight-threatening diseases.

The vitreous is a largely acellular extracellular matrix, consisting >98% of water, with collagen fibres (types II, IX and XI) maintaining the structure, with glycosaminoglycans, mainly hyaluranon, and other macromolecules including fibrillin and opticin, maintaining the interfibrillar separation and preventing collapse of the gel [1]. It serves to act as a transparent medium for light to pass unimpeded to the retina, and provides metabolic modulation for the lens, as evidenced by the progression of cataract following surgical removal of the vitreous [2]. With aging, there is liquefaction of vitreous with aggregation of collagen fibrils [3, 4], which may be related to elevated levels of proteolytics enzymes in the vitreous [5]. It has previously been considered that the liquefaction of vitreous led to PVD when this fluid gained access to the retrohyaloid space, but this does not explain the variations in state or progression of PVD seen in clinical practice, nor the separation of the PHM from the retina in eyes in which the vitreous body has already been removed [6, 7]. Liquefaction may therefore be a predisposing factor for PVD, a necessary initial step, or they may be two unrelated age-related correlates.

There is now a considerable body of research demonstrating that the PHM is the structure enveloping the posterior aspect of the vitreous gel, and separates from the internal limiting membrane (ILM) of the retina during PVD, where it becomes visible using off-axis slit-lamp biomicroscopy [8–10]. It is recognised surgically during vitrectomy either in its already detached state or sometimes requiring surgical separation from the retinal surface. Light microscopy and immunohistochemistry have demonstrated that the PHM stains strongly for type IV collagen [9, 10], known also to be present in the ILM [11] and other basement membranes [12] and can have areas of reduplication and focal schisis. It follows therefore that immediately prior to PVD, the PHM must form part of the ILM. There is also an associated population of cells, laminocytes, which can be seen clinically studying the detached PHM with slit-lamp biomicroscopy. Electron microscopy studies have also shown these cells associated with the PHM [9].

There is, however, no consensus regarding from where the PHM originates during PVD, or whether it is indeed a structure distinct from peripheral vitreous gel matrix (‘vitreous cortex’). The processes that lead to separation of the PHM from the ILM of the retina are not understood. It is unclear what the role of the cells associated with the PHM is.

This study investigates the ultrastructure of the PHM in post-mortem eyes with PVD using transmission electron microscopy (TEM) and scanning electron microscopy (SEM), and compares the findings with those eyes in which the vitreous was attached.

## Methods

Collection and use of human tissue in this study was granted prospective approval by the research ethics committee. Relatives of recently deceased patients in a hospital mortuary were approached for written informed consent for organ donation for research purposes. Eyes with previous retinal surgery were excluded. If consent was granted, the eyes were enucleated and fixed in 2.5% glutaraldehyde; 3 mM tannic acid; 80 mM sodium cacodylate.

An 18 mm trephine was centred on the optic nerve and a button of sclera, choroid and retina removed, and presence or absence of PVD identified as previously described [13]. In eyes with PVD, a specimen of PHM along with the vitreous gel on its anterior face was taken from the vitreous surface, and where possible at the anterior extent of PVD with its attachment to the retina. In eyes without PVD, a specimen of the exposed convex vitreous surface along with the vitreous gel on its anterior surface was taken.

### SEM

Samples were fixed in 2.5% glutaraldehyde in 0.08 M cacodylate buffer. They were washed 3 times in phosphate buffer and then osmicated with 1% osmium tetroxide in ddH2O for 1 h. Samples were then washed 3 × 10 min in ddH2O and dehydrated with a series of 30%, 50%, 70%, 90% and 3 × 100% alcohol. The samples were then immersed 2× in dry methanol and 2× in hexamethyldisilazane (reagent grade >99%, Aldrich chemicals) and allowed to dry in a fumehood.

The specimen was placed onto an aluminium stub using a conductive carbon disc. The sample was further adhered to the stub using silver paint (Agar). It was then dissected to reveal the internal structure. Then coated with 2 nm platinum in a Cressington sputter coater.

The sample was imaged on a Zeiss Sigma VP SEM using the in-lens detector.

### TEM

Samples were fixed in 2.5% glutaraldehyde in 0.08 M cacodylate buffer. They were washed 3 times in phosphate buffer and then osmicated with 2% osmium tetroxide in ddH2O for 1 h. Samples were then washed 3 × 10 min in ddH2O and dehydrated with a series of 30%, 50%, 70%, 90% and 3 × 100% ethanol, then 2× in propylene oxide. They were left overnight in 50% propylene oxide/araldite resin and then overnight in 100% resin. A further exchange into 100% resin was performed before they were baked in an oven (60°).

For feature identification, 650 nm sections were cut from araldite blocks using a Leica Ultracut. They were then stained with 0.05% aqueous Toluidine Blue in 2% Borax. They were then imaged on a Leica EVOS slide scanner.

Samples were sectioned on a Leica ultramicrotome (70 nm), sections placed onto copper grids and stained with Reynold’s lead citrate. Images were collected on a Jeol 1400 + TEM fitted with a Gatan Orius camera.

## Results

Twelve eyes were collected from six patients with age of death ranging from 75 to 100 years (mean 85 years). There was PVD in 7 of 12 eyes (58%), a similar incidence to previous studies [4, 8], and PHM was isolated in all of these eyes. One of these eyes had an incidental finding of an operculated retinal tear.

**Fig. 1:**
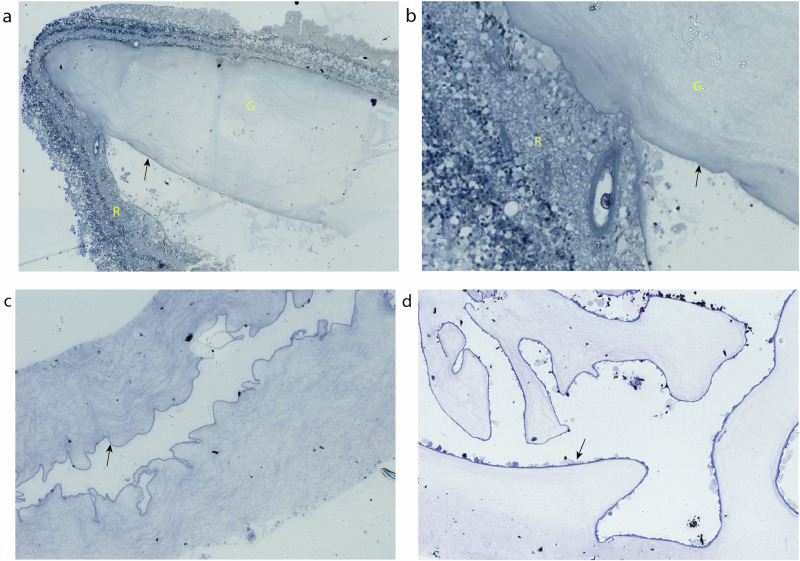
Light microscopy images from eyes with and without posterior vitreous detachment. **a** Low magnification light microscopy image of toluidine blue stained section of vitreous and retina at anterior aspect of posterior vitreous detachment showing vitreous gel fibres (G), posterior hyaloid membrane (arrow) and retina (R). **b** Higher magnification image showing the vitreoretinal interface at the anterior extent of posterior vitreous detachment. **c** Low magnification light microscopy image of toluidine blue stained section of vitreous and posterior hyaloid membrane isolated from eye with posterior vitreous detachment, demonstrating vitreous gel with smooth encapsulating membrane (arrow). **d** Vitreous and membrane isolated from eye where vitreous was still attached at time of dissection, demonstrating residual debris on retinal surface of membrane (arrow).

Eyes with PVD had an identifiable anterior limit to the separation from the retina, where the PHM merged with the ILM [Fig. 1a, b].

**Fig. 2:**
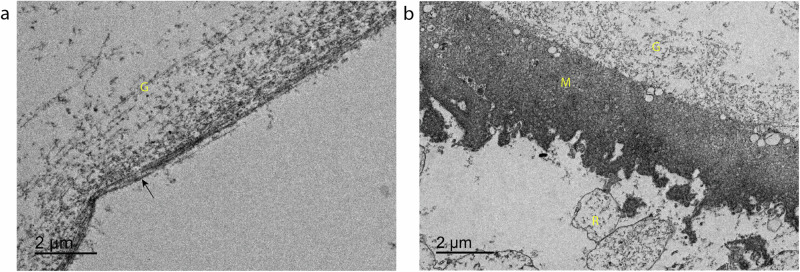
Transmission electron microscopy comparing eyes with posterior vitreous detachment versus with attached vitreous. **a** Transmission electron microscope (TEM) image of vitreous and posterior hyaloid membrane isolated from an eye with posterior vitreous detachment, demonstrating vitreous gel fibres (G) and smooth membrane surface (arrow). **b** TEM image of vitreous and membrane isolated from eye where vitreous was still attached at time of dissection, demonstrating vitreous gel fibres (G), membrane (M) of very similar morphology to internal limiting membrane, and residual debris on the non-vitreal surface, presumed of retinal origin (R).

In eyes with PVD, the PHM is visible as a dark band on the toluidine blue stained light microscopy [LM] images, with a smooth border on the non-vitreous side [Fig. 1c]. This is in contrast to the samples without PVD, in which a similar membrane appears but with considerable debris on the retinal-facing surface [Fig. 1d]. TEM imaging reveals that in eyes with PVD, the PHM is a smooth laminar surface between the vitreous gel collagen fibres and the empty retinal aspect [Fig. 2a]. This is in contrast to the eyes without PVD, where the TEM shows the ILM encasing the vitreous gel collagen fibres with artefactual retinal material still attached to its undersurface [Fig. 2b].

**Fig. 3:**
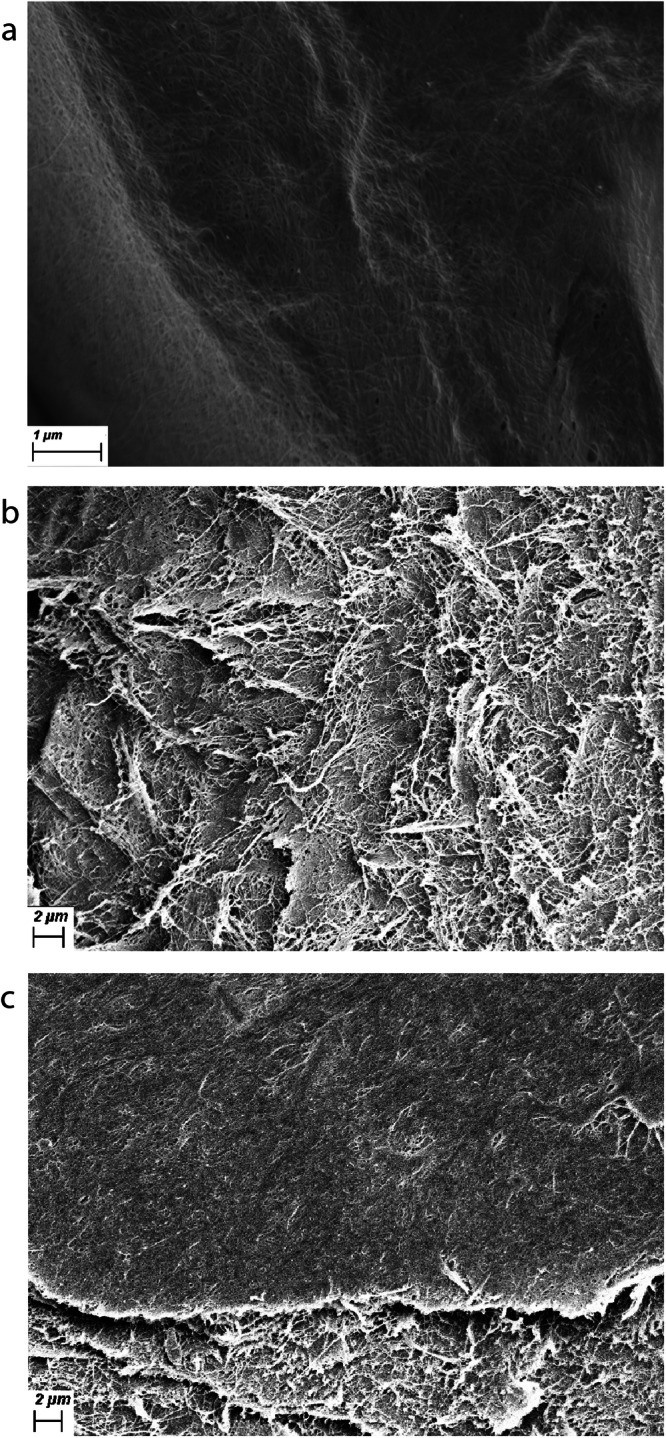
Scanning electron microscopy of posterior hyaloid membrane versus vitreous gel. **a** Scanning electron microscope (SEM) image of the surface of the posterior hyaloid membrane (PHM) isolated from an eye with posterior vitreous detachment (PVD), demonstrating a smooth membrane with fine lacy fibres. **b** SEM image of vitreous gel fibres showing a disorganised array of fibres (collapsed by dehydration in processing for SEM). **c** SEM image of a sample from an eye with PVD showing the smooth PHM with the disorganised vitreous gel fibres underneath.

**Fig. 4:**
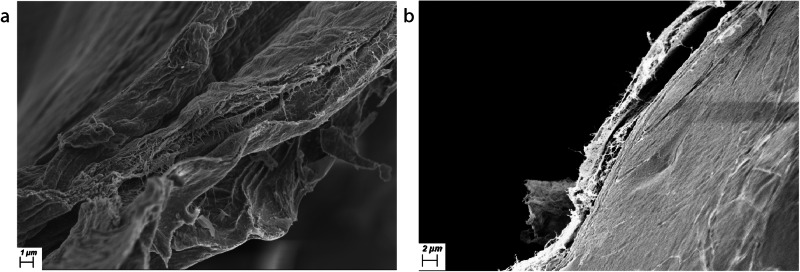
Scanning electron microscopy orthogonal to the surface of the posterior hyaloid membrane. **a**, **b** Scanning electron microscope (SEM) image of the cut edge of the posterior hyaloid membrane isolated from an eye with posterior vitreous detachment, demonstrating the multilayered structure.

On SEM, the PHM appears as a smooth flat sheet comprising fine interwoven fibres [Fig. 3a]. This in contrast to the disorganised fibres seen in vitreous gel [Fig. 3b, c]. With an orthogonal view, the PHM appears to have a laminar structure [Fig. 4a, b].

**Fig. 5:**
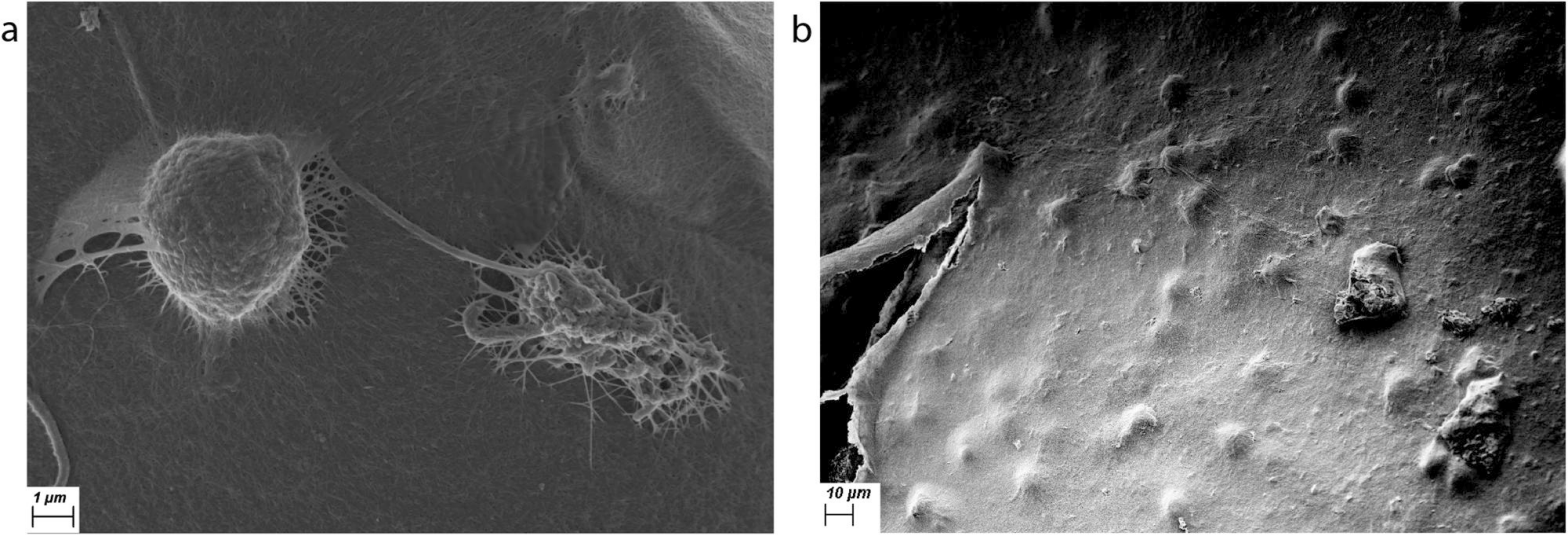
Scanning electron microscopy of laminocytes. **a** Scanning electron microscope (SEM) image of two cells, one collapsed, on the surface of the posterior hyaloid membrane (PHM) isolated from an eye with posterior vitreous detachment (PVD) demonstrating strands emanating from the cell into the membrane, creating a laminar structure. **b** Scanning electron microscope (SEM) image of several cells embedded within the PHM isolated from an eye with PVD.

Cells were identified in six of the seven samples of isolated PHM, often with strands stretching from the cells into a multilayered membrane [Fig. 5a], and sometimes embedded within the membrane [Fig. 5b].

## Conclusion

This study adds novel EM findings to previous immunohistochemical studies of the PHM isolated from eyes with PVD and demonstrates the ultrastructural differences from artefactual separation of the ILM from the retina during post-mortem dissection [8–10, 14]. The apparent debris on the retinal side of vitreous surface of eyes in which the vitreous was attached correlates with the TEM appearance of ILM plus residual vesicular material, presumably from retinal Müller cells. This demonstrates that when there is a macroscopic appearance of attached vitreous following removal of a posterior scleral button, the dissection process has artefactually cleaved the ILM from the retina (rather than the PHM from the ILM). Conversely, when there is a macroscopic appearance of PVD, the isolated PHM is thin and smooth with no debris and no ultrastructural similarity to ILM. This is reminiscent of Sebag’s 1991 study [15] of the vitreoretinal interface in different ages, in which there was a similar finding in many eyes of young patients of ILM remaining attached to the vitreous that had been dissected away from the retina, as the vitreous is highly likely to be attached in young patients of this age group. The investigators in this current study also noticed that in eyes with PVD it was possible to section the retina and acquire TEM images of (degraded) retina with ILM, but in the eyes without PVD (i.e. the ILM had been stripped from the retina during dissection), the retinal samples were too unstable for processing and no TEM imaging was possible, presumably due to the lack of structural stabilisation from the removed ILM. The dissection and processing methods used have been shown in previous studies to correctly identify the post-mortem macroscopic post-dissection appearance of PVD vs no PVD in correlation with antemortem clinical findings [8–10].

There is clear ultrastructural difference between the PHM and the vitreous gel fibres, supporting recent research showing the PHM as a specific structure distinct from the peripheral vitreous gel matrix (‘vitreous cortex’). This also explains the widely recognized clinical phenomenon that a ‘PVD’ with associated retinal tear can still occur years after all vitreous has been surgically removed [7]. The en face appearance of PHM is strikingly similar to the appearance of ILM [15–18], and it has been clearly shown that both are composed of type IV collagen [8, 10], among other supporting molecules such as laminin, fibronectin, and complex carbohydrates [19], supporting the concept that prior to PVD, the PHM forms from the separation of the inner layer of ILM. It is notable that, in keeping with other basement membranes, the ILM thickens with age. Heegaard [20] measured ILM thickness with TEM in post-mortem eyes across human lifespan from foetal eyes to those in the tenth decade. Although there was no reported statistical correlation with increasing age beyond an increase between foetal and adult eyes, this was analysed assuming a continuous increase over time. However, reappraising the original data the macula thickness increases from a median of 68 nm in the foetal eye, median 1768 nm in the third decade, up to a maximum thickness in the fourth and fifth decades (median 3400 and 3060 nm, respectively), but then decreased in the sixth and seventh and ninth decades (median thickness 2924 nm, 2448 nm and 2584 nm, respectively). This can be explained if the basement membrane thickened with age as expected but PVD had occurred in a proportion of the older group, with the PHM removing an anterior layer of the ILM, resulting in a reduction in thickness.

There are cells embedded within the PHM, which may be secreting fibres into the membrane, creating a smooth close knit laminated structure. It is likely that these previously described laminocytes [9, 13, 21] play an active role in the process of PVD and associated pathological vitreoretinal conditions including epiretinal membrane, macular hole and retinal detachment. In ERM they are associated with areas of reduplication of ILM, strongly expressing type 4 collagen, and have been associated with focal contraction of PHM in PVD [21]. Laminocytes are known to express GFAP, which was originally considered a specific protein marker for glial cells, although there is evidence for its presence in other cell types including fibroblasts [22–25]. Laminocytes have also been shown to not stain with antibodies to CRALB or S100B as glial cells or Müller cells would be expected to, and laminocytes stain positively with antibodies to MHC Class II and CD68, suggesting they may be a macrophage subpopulation [14]. Laminocytes therefore represent a specific cell group, with similarities to, and differences from, both glial and macrophage populations.

Recent in vivo imaging has captured these cells laying on the vitreoretinal interface in attached vitreous, variously described in OCT studies as ‘vitreous cortex hyalocytes’ [26, 27], or macrophage-like cells just above the ILM [28], and in adaptive optics studies, again as ‘vitreous cortex hyalocytes’ [29], or ILM macrophages [30]. Due to their association and orientation across sheets of tissue, and their ability to synthesise new basal lamina, we favour the original term ‘laminocyte’ as best describing and unifying these distinguishing cellular characteristics

Many further questions remain about the PHM. Given the laminar nature of the PHM, it is possible that more than one ‘split’ could develop in the anterior ILM, which is consistent with the reduplication that has previously been demonstrated in both the PHM [9] and ILM [21, 31]. It would be interesting to compare the anteroposterior strength of the ILM and how that may vary with thickness, and therefore its propensity to split and detach, versus its shear stress i.e. ability to tear in the same plane as the retina, and the propensity for PVD to ‘leave behind’ areas of PHM and vitreous on the retinal surface—another feature sometimes described as ‘vitreoschisis’. It may be that the thicker the ILM becomes, the less stable it is against a laminar split, and may explain why PVD arrests at or beyond the equator where the ILM in thinner. It could be that in areas of variable lamination or thickness, the cleavage plane is able to enter the retina, which then causes a retinal tear.

The activity and function of the laminocytes associated with PHM require further investigation. Our research group is using a transcriptomics approach to analyse and interrogate the RNA transcription profile of laminocytes both in bulk and spatial analysis in PVD and other vitreoretinal conditions in the hope of identifying biological pathways involved in the development of PVD that could be targets for intervention to enable the induction, inhibition or modification of PVD to reduce the burden of vitreoretinal disease in the future.

## Summary

### What was known before

- Posterior vitreous detachment is the separation of the posterior hyaloid membrane from the retina, and is a significant factor in vitreoretinal disease.
- Immunohistochemical studies demonstrate that a major component of the posterior hyaloid membrane is type IV collagen, similar to the internal limiting membrane, and that there is a distinct associated cell population.

### What this study adds

- The posterior hyaloid membrane appears to form from a separation of an anterior part of the internal limiting membrane. The posterior hyaloid membrane is laminar in structure, with cells on the surface or embedded within spreading fibres into the membrane.

## Data Availability

The data generated during the current study are available from the corresponding author on reasonable request.
